# Comprehensive Care after Myocardial Infarction (CCMI): Long-Term Investment in the Health of Polish Citizens

**DOI:** 10.3390/ijerph19127518

**Published:** 2022-06-20

**Authors:** Grzegorz Kubielas, Paulina Hydzik, Łukasz Rypicz

**Affiliations:** 1 Department of Nursing and Obstetrics, Faculty of Health Sciences, Wroclaw Medical University, 51-618 Wrocław, Poland; paulina.hydzik@umw.edu.pl; 2 Department of Health Care Services, Central Office, Polish National Health Fund, 02-528 Warsaw, Poland; 3 Department of Population Health, Faculty of Health Sciences, Wroclaw Medical University, 50-372 Wrocław, Poland; lukasz.rypicz@umw.edu.pl

**Keywords:** comprehensive cardiac care, myocardial infarction, CCMI model, cardiac rehabilitation, secondary prevention, cardiovascular disease prevention

## Abstract

The comprehensive care model after myocardial infarction (CCMI, in Polish: KOS-Zawał) has been in effect continuously since October 2017. Within the bundle of services financed by the Polish National Health Fund (NHF), patients receive a diagnosis, conservative and invasive treatment, early cardiac rehabilitation and follow-up visits for 12 months. The existing model of managing patients after myocardial infarction (MI) implements all crucial aspects of care recommended by the European Society of Cardiology (ESC), emphasised many times. The purpose of this paper was to report and describe the course of the implementation of the unique concept—CCMI model, including the scope of the introduced changes and the implementation and structural evaluation of its effects over the period 2017–2021. Our preliminary study reported that the CCMI programme reduces the risk of patient death in the first year after MI by 29%. Furthermore, the authors point out the strict cause and effect relationship between the cardiovascular disease prevention programme since 2004 as the key instrument for the primary systemic prevention implemented outside the CCMI model.

## 1. Introduction

According to 2016 data, cardiovascular diseases (CVDs), including coronary heart disease, accounted for more than 43% of deaths in the general population [1]. In 2019, more than 102 thousand persons were hospitalised for acute coronary syndromes (ACS). Furthermore, the observed increase in hospitalisations for myocardial infarction (MI) within the last six years reached 9% [2].

However, it should be noted that hospital mortality from MI from 1980 to 2016 decreased three times from 22% to 6%. This successful effect was achieved by significant progress in MI treatment quality and effectiveness [1]. Furthermore, increasing funding for cardiology areas, including primarily for invasive cardiology, enabled the establishment of the densest network of hemodynamic laboratories in Europe. This formed the basis for developing the interventional procedure model, including the primarily large-scale performance of percutaneous coronary interventions (PCI) that became the important pillar of therapy in this group of patients [3,4].

On the other hand, the negative trend in 12-month mortality involving 10.1% of patients who were discharged from the healthcare facilities after MI should be taken into account. The causes of such a significant mortality rate include, among others, failure to introduce healthy lifestyle patterns by the patients, failure to adhere to therapy, and also disturbed or insufficient access to specialist cardiac care, including early rehabilitation [5].

Before the solutions were introduced in the autumn of 2017, Poland had no modern organisation system and implementation of early cardiac rehabilitation and secondary prevention of CVDs. In 2013, Jankowski et al. [6] demonstrated that only 23% of patients were referred to facilities with comprehensive rehabilitation after MI. According to the Polish National Health Fund (NHF) data, in 2016, only 3% of the patients among nearly 126 thousand ACS cases received cardiac rehabilitation within 14 days following hospital admission [1].

Another issue of concern affecting the increased non-hospital mortality was the frequency of specialist medical consultations (cardiology consultation within the specialist outpatient care). According to the Silesian Cardiovascular Base data, only 66% of all patients after MI obtained cardiology consultation, while the median time from discharging from the hospital to the first consultation was four months [3].

According to Statistics Poland data, failure to introduce the essential changes to secondary prevention of acute coronary syndromes and their treatment could lead to 51% of deaths from CVDs in 2050 [7]. Moreover, our preliminary study reported that the CCMI programme reduces the risk of first-year mortality after MI by 29%. Moreover, we reported a lower mortality rate in the CCMI group compared with the non-CCMI group regarding in-hospital and 30-, 180-, and 365 days post-hospitalization, respectively: 0.19% vs. 6.55%; 0.80% vs. 8.39%; 2.92% vs. 10.74%; and 6.35% vs. 13.40%. Overall survival analysis showed a significantly lower probability of death in the CCMI patients [8].

This situation required the implementation of deliberate and well-organised measures to decrease non-hospital mortality from MI and the introduction of a coordinated treatment and rehabilitation system. Therefore, this paper established a comprehensive management CCMI model covering all patients after MI, including diagnosis, treatment, rehabilitation, and follow-up visits.

## 2. Genesis of the Programme

The factual basis for the commencement of studies on developing the comprehensive care model was a joint statement of the Polish Cardiac Society and the Agency for Health Technology Assessment and Tariff System (AOTMiT). The experts demonstrated that treating patients with MI cannot be completed at discharge from the hospital. In the 2013 report, the Polish Cardiac Society revealed the results, in which coverage of only half of patients after MI would lead to avoidance of 2200 deaths and 15,000 hospitalisations due to cardiac reasons per annum. To significantly decrease the mortality rates of patients after MI, the continuation of treatment in the scope of secondary prevention is required [6].

The analyses carried out together with the AOTMiT regulations and European Society of Cardiology (ECS) recommendations formed the basis to establish the model rules of care for patients after MI. The significant role of risk factor control, which directly affects longer life expectancy and better quality of life, was emphasised. According to experts, invasive treatment, comprehensive rehabilitation, cardiology education, and specialist care should be included in the programme [5,6].

The programme of comprehensive care after myocardial infarction (CCMI, in Polish: KOS-Zawał) aimed to provide the patients after MI with comprehensive, easily accessible, specialist and yearlong care (12 months after treated MI episode). The purpose of this care also includes better access to physiotherapy, nursing, psychology and dietetics specialists (according to recommendations). With the cooperation of an interdisciplinary team, the aim was to implement a multi-stage, affordable model based on up-to-date medical knowledge and information on patients, focusing mainly on cardiac rehabilitation and secondary prevention (correcting undesired behaviours in CVDs). The CCMI model should minimise mortality and maximise treatment opportunities for patients after MI, therefore leading to their full recovery, including a return to their social roles such as their occupational activities [9].

## 3. Qualification Criteria

In accordance with the assumptions, patients with the following diagnoses are eligible for the service (according to ICD-10) [10]: I21.0 Acute transmural MI of the anterior wall; I21.1 Acute transmural MI of the inferior wall; I21.2 Acute transmural MI of other sites; I21.3 Acute transmural MI of the unspecified site; I21.4 Acute subendocardial MI; I21.9 Acute MI, unspecified; I22.0 Subsequent MI; I22.1 Subsequent MI of the inferior wall; and I22.9 Subsequent MI of unspecified site.

## 4. Scope of Service

**Figure 1:**
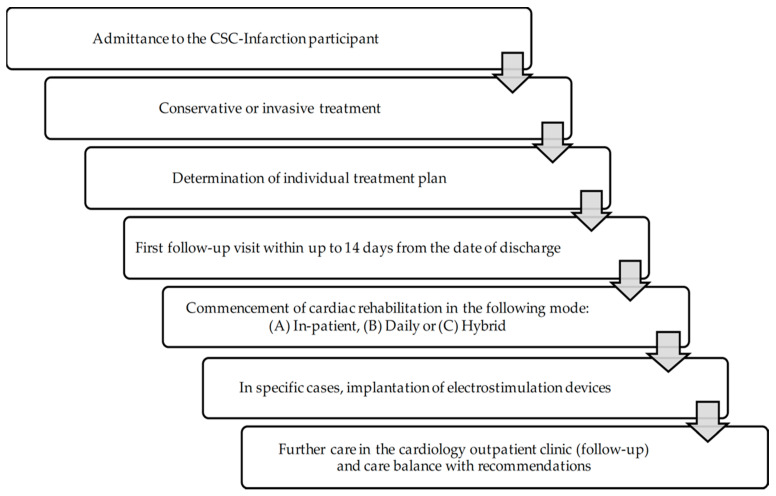
A schematic patient path within the CCMI model.

CCMI consists of the provision of individual services according to the individual patient’s condition (see Figure 1):

(1) Conservative or invasive treatment or invasive diagnosis of MI;
(2) Implantation of a relevant implantable cardioverter-defibrillator (ICD) or cardiac resynchronisation therapy defibrillator (CRT-D);
(3) Coordination (follow-up) visits within up to 14 days from discharge, during which the following tests and examinations are performed: electrocardiographic examination, laboratory tests (complete blood count, blood potassium level, blood creatinine level, and C-reactive protein level—CRP).

## 5. Programme Launching

The innovative CCMI programme was launched on 17 October 2017, dedicated to all patients after MI (inclusion criterion according to ICD-10). The introduction of the CCMI model aimed to ensure multidimensional benefits. Its profitability was to cover the health effects of patients and the financial effects of the healthcare system.

In order to conclude the contract, the healthcare provider must meet numerous formal requirements, including, among others, the presence of the following wards in its organisational structure: (1) cardiology ward; (2) radiosurgery or haemodynamic laboratories, as well as electrophysiology laboratories, e.g., based on subcontracting; (3) cardiac surgery ward; (4) in-patient cardiac rehabilitation ward; (5) specialist outpatient cardiology clinic; (6) 24/7 telephone contact for the patients under the programme and 24/7 consultations [11].

To provide the CCMI services, the healthcare provider (medical entity) appoints the coordinator to supervise the treatment plan and support the patient’s recovery period. The coordinator’s duties primarily include scheduling the appointments, monitoring the quality of each treatment stage and supervising the documentation. Initially, the programme was composed of 4 modules where:

- ***Module I*** covered hospitalisation with conservative/invasive treatment and invasive diagnosis. The first component also included preparing the patient care plan and scheduling the coordinating appointment that completed this module,
- ***Module II*** covered cardiac rehabilitation, which could be performed in a day, in-patient or hybrid mode. The rehabilitation component should take place for 14 days from hospital discharge after complete coronary revascularisation,
- ***Module III*** covered electrotherapy, which can be performed out of turn. In addition, it enables the implantation of CRT-D or ICD,
- ***Module IV*** included specialist cardiac care lasting 12 months from MI event.

As part of their healthcare, the patient had access to consultations every day of the week and access to services ensuring diagnostics, which, if required by the patient’s condition, had to be performed on a 24/7 basis in the cardiology ward. Outpatient visits within the CCMI were unlimited; however, they were required to take place at least three times within 12 months of the programme duration. This stage ended with a follow-up visit in the form of check-up care that included specialist consultations and diagnostics [12]. According to the preliminary tariff estimations of the Agency for Health Technology Assessment and Tariff System, the CCMI programme will cover 38% of the patients. However, the programme covered less than 8% at the initial implementation stage [5,9].

**Figure 2:**
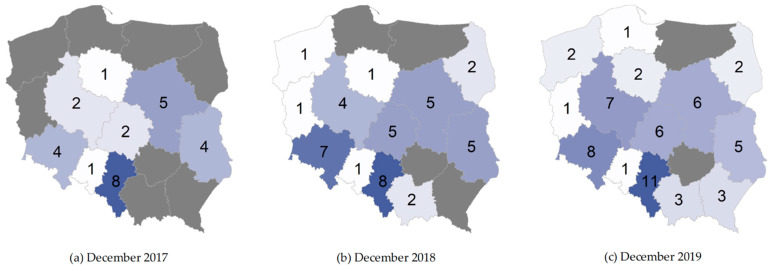
Number of health centres providing services under the CCMI programme (December 2017–December 2019).

**Figure 3:**
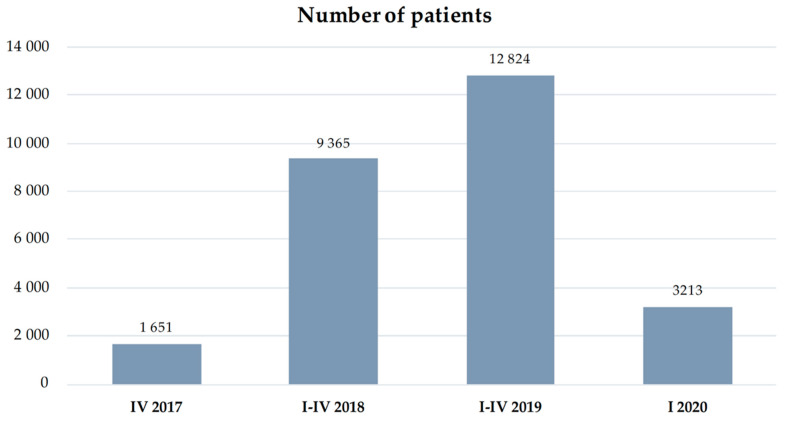
Number of patients provided with services under the CCMI programme for the first time (IV 2017–I 2020).

**Figure 4:**
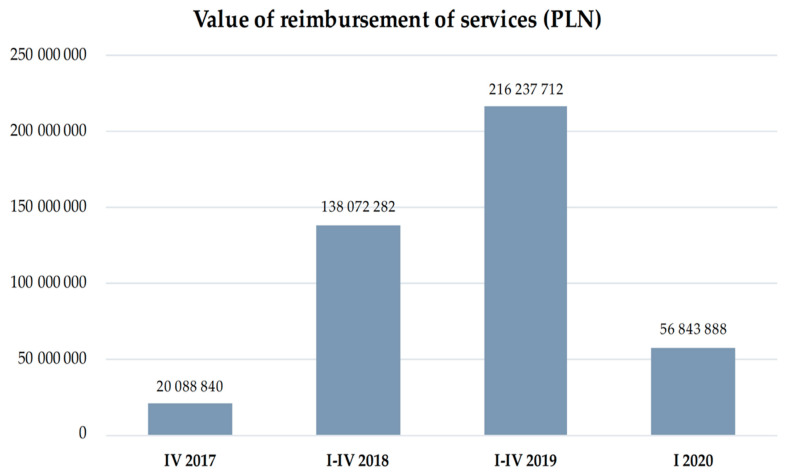
Value of reimbursement of services (in PLN million) provided within the CCMI programme (IV 2017–I 2020).

Distribution of the healthcare facilities in the country demonstrated significant irregularity. It should be noted that the programme was launched in the 4Q 2017. According to 2017 data provided by the Department of Healthcare Services of the NHF, the CCMI programme was implemented by 31 providers and 47 providers a year later. The value of concluded contracts for the programme implementation in 3 months of 2017 amounted to PLN 32.5 million, while in 2018, the total amount of contracts reached nearly PLN 140 million [13]. The most CCMI programme funds were allocated to the cardiology centres operating in the Śląskie Region (PLN 78.2 million), with the greatest number of patients benefiting from care [14]. It should be stressed that, due to the lack of regionalisation, patients with acute MI can be referred to any facility (see Figure 2, Figure 3 and Figure 4).

The key question is how to explain the poor interest in the programme at its initial stage, which applied to medical centres and patients (in 2017–2018). One of the crucial factors that affected potential participants’ poor willingness to join the programme was inequalities in remuneration for implementing the programme by the university institutes and hospitals. The value of contracts signed with the NHF by these institutions increased by only 5%, and by city and county hospitals by 15%. Another negative factor was the inability to settle the implantation of electrical devices, such as the cardiac resynchronisation system, under the CCMI.

An issue of concern was also the coordinating visit, which was supposed to be scheduled on the third day after the patient’s discharge. The criteria for earlier withdrawal from care under the programme also raised many doubts [15,16]. At the same time, it should be noted that the issue of accessibility of the CCMI participants is identical to other comprehensive care programmes implemented in recent years in Poland. The lack of interest from potential participants stems from the need to comply with the additional requirements necessary to provide these services and, more importantly, in a manner enabling no derogations. Since the monitoring and settlement processes are strictly correlated, failure to comply with the deadlines by the participant (laid down in the Ordinance) or withdrawal of the patient from the given module results in losses in settlement to the participant. In addition, the participant is forced to continuously monitor the performance indicators, which, as shown by experience (since 2017), is problematic.

## 6. Improving Programme Implementation

In 2017, the new order of NHF President [17] specified the terms and conditions of concluding and implementing the contracts for hospitalisation: comprehensive services [11]. The amendment came as a result of the number of issues requiring changes in settlement of these services observed after the first year of their provision within the CCMI. The healthcare providers reported, among others, the following issues:

- The need to introduce clear and transparent provisions on early withdrawal from CCMI or transfer of the patient to other centres with a signed contract for the provision of these services;
- Absence of a correction factor for cardiology in institutes, such as in the case of implementing the contract for hospital services;
- The need for remuneration for the entities with an in-house rehabilitation ward (analogically to the coronary artery bypass grafting at the cardiology ward in the in-house organisational structure);
- Enabling settlement of correction factor at the time of issuing the certificate of cardiac contraindications to work;
- The problems with settling the services from the catalogue of services to be summed up and maintained at the Anaesthesiology and Intensive Care Unit;
- Enabling all Diagnosis Related Groups (DRGs) in the scope of electrotherapy to be implemented within CCMI.

Detailed arrangements include: the introduction of the factor of 1.1. for services provided at the daily cardiac rehabilitation ward in the organisational structure when settling the daily rehabilitation services and factor of 1.1 for the institutes, on the same principles as in the case of implementing the contract for hospital services, as well as adding the group E31 Implantation/replacement of single-chamber pacemaker, E32 Implantation/replacement of dual-chamber pacemaker and E33 Implantation/replacement of CRT-D. In addition, the structure of Appendix 1k was amended, including replacing the modules with division into treatment, rehabilitation and follow-up, and separate settlement of coordinating–follow-up visits.

As a result, the coordinating (follow-up) visit date was amended up to 14 days following hospital discharge. In addition, the healthcare providers providing the CCMI service were obliged to report and assess the quality parameters laid down in the Ordinance. At the same time, due to the fact that no current number of patients requiring daily cardiac rehabilitation provided by the healthcare providers with cardiac rehabilitation ward in their organisational structure is known, the financial effect of introducing the new correction factors is difficult to estimate.

In addition, the modules were replaced with the CCMI structure in three areas:

(1) TREATMENT covers hospitalisation of the patient with MI involving:
  (a) Invasive diagnosis of MI (coronary angiography) or conservative treatment;
  (b) Invasive treatment covering, among others, complete coronary revascularisation or cardiac surgery (adequately to the clinical condition of the patient);
  (c) Single-stage revascularisation during hospitalisation for MI;
  (d) Double-stage revascularisation that can be received at the first hospitalisation for MI or divided into two hospitalisations (first for MI and the second planned hospitalisation);
  (e) Electrotherapy, including:
    - Implantation/replacement of single-chamber pacemaker;
    - Implantation/replacement of double-chamber pacemaker;
    - Implantation/replacement of cardiac resynchronisation therapy (CRT) device;
    - Implantation/replacement of ICD;
    - Implantation/replacement of CRT-D;
    - Implantation/replacement of S-ICD after assessment of ejection fraction (EF) in the patient within the individual treatment plan prepared during hospitalisation by the cardiologist (cardiac rehabilitation and cardiology outpatient clinic).
  (f) Patient treatment plan should be enclosed in the patient’s medical record;
(2) REHABILITATION covers:
  - Cardiac rehabilitation or hybrid cardiac telerehabilitation in in-patient conditions;
  - Cardiac rehabilitation or hybrid cardiac telerehabilitation in the centre or daily ward under the eligibility criteria and conditions required to provide such rehabilitation laid down in Appendix 5 to the Rehabilitation Ordinance.
(3) FOLLOW-UP covers:
  - Coordinating (follow-up) visits;
  - Specialist cardiac care within 12 months from MI according to the individual treatment plan (including consultations covering eligibility for implantation of completely automatic implantable cardioverter-defibrillator (ICD), CRT-D or S-ICD and follow-up of implantable devices); the number of consultations and their frequency should depend on the clinical condition on the patient;
  - Specialist consultation to complete care within the CCMI.

### 6.1. Withdrawal

The CCMI is withdrawn earlier when:

(a) No subsequent care component was provided within the time frame specified in the Hospital Ordinance, Outpatient Ordinance or Rehabilitation Ordinance;
(b) There is a need for third or subsequent revascularisation;
(c) Another MI event took place;
(d) The patient continued treatment with another healthcare provider with a signed contract for CCMI;
(e) The patient died.

### 6.2. Financing

All healthcare services offered to the patient under the CCMI model and resulting from the individual treatment plan are financed from public funds. The healthcare service provider, having fulfilled the conditions required for the provision of services under the CCMI program, including in-patient treatment, cardiac rehabilitation and outpatient specialist care, enters into an agreement with the NHF and receives dedicated funds for their provision. Due to the nature of these services, the MI treatment in the Polish healthcare system is financed without a limit and during the reviewed period 2017–2020, there were no issues reported in this regard.

After fulfilling certain conditions, healthcare service providers receive additional remuneration through the introduction of correction coefficients and payment for the performed procedures (provided benefits). The introduced mechanism is intended to reward the provision of coordinated and comprehensive care by the main provider on specified dates. The main assumption of the adopted solution is to reward the quality of provided services and the evaluation activities undertaken; each of the healthcare service providers (implementors) of the CCMI program is obliged to evaluate the effects of comprehensive care annually.

Financing the individual stages of comprehensive care provided adequately to the clinical needs of patients covers:

(1) In the area of treatment:
  (a) Invasive diagnosis, conservative treatment, covering following the clinical condition of the patient, coronary revascularisation or coronary artery bypass grafting provided within hospitalisation: settled within the DRG from the catalogue of comprehensive services;
  (b) Additional services settled as unitary products listed in the catalogue of services (Appendix 1c and 1ts to the Hospitalisation Ordinance): to be summed up with relevant DRG from the catalogue of comprehensive services,
  (c) Preparation of the individual comprehensive treatment plan after MI: settled as the product from the catalogue of comprehensive services to be summed up with the relevant DRG from this catalogue,
  (d) Electrotherapy: settled within the relevant DRG from the catalogue of comprehensive services;
(2) In the area of rehabilitation:
  (a) In-patient cardiac rehabilitation: settled within the CRMI group (cardiac rehabilitation as part of comprehensive care after myocardial infarction in an in-patient setting) from the catalogue of comprehensive services;
  (b) Cardiac rehabilitation in the centre at the daily ward and hybrid cardiac telerehabilitation provided at the place of stay of the patient: settled as a person/day within the products from the catalogue of comprehensive services.
(3) In the area of follow-up:
  (a) Coordinating (follow-up) visit: settled as the product from the catalogue of comprehensive services;
  (b) Specialist cardiac care within 12 months from MI covering:
  (c) At least three consultations within the period of CCMI coverage: settled on a lump-sum basis after provision within the product from the catalogue of comprehensive services;
  (d) Specialist consultation along with care balance in the patient completing the care under CCMI (examinations and assessment of clinical condition): settled within the settlement product: “Specialist cardiac care–care balance” from the catalogue of comprehensive services [10].

### 6.3. Benefits

In order to give providers a premium under the CCMI program, the President of the NFZ introduced correction coefficients for services listed in the comprehensive benefits catalogue, provided that the following conditions are met:

(a) In the case of provision of CCMI services at the cardiology ward in the organisational structure, operating in the 24/7 mode with a separated emergency unit, when settling the services of coronary artery bypass grafting within the groups: E04, E05, E06 and E07, the value of these settlement products is corrected with the use of the correction factor of 1.2;
(b) In the case the patient starts cardiac rehabilitation within 14 days from discharge, settlement of provided rehabilitation is corrected with the use of the correction factor of 1.1;
(c) In the case of the provision of CCMI services at the daily cardiac rehabilitation ward in the organisational structure, when settling the daily rehabilitation services, the value of these settlement products is corrected with the use of the correction factor of 1.1;
(d) If a professionally active patient after MI will receive a medical certificate issued by the attending physician of no cardiovascular contraindications to take up/perform a job up to the fourth month from discharge, the value of settlement products (laid down for treatment and follow-up), is corrected with the use of correction factor of 1.1 after issuing of this certificate;
(e) If the patient received all services specified in the individual treatment plan within 12 months under the CCMI assumptions: the value of settlement products (laid down for treatment and follow-up), is corrected with the use of a correction factor of 1.15 when settling the care balance;
(f) For the healthcare providers being:
  - The institutes referred to in Article 3 of the Act of 30 April 2010 on research institutes (Journal of Laws of 2019, item 1350);
  - Medical entities established and operated by a medical university in the meaning of Article 2 (1) (13) of the Act of 15 April 2011 on medical activity;
  - Medical entities established and operated by the State Treasury represented by the minister, involved in post-gradual education of physicians;
  - Medical entities providing a medical university with access to organisational units necessary to perform pre- and post-graduate education in medical professions based on the agreement referred to in Article 89(4) of the Act of 15 April 2011 on medical activity (the entity is obliged to present the agreement to the competent branch of the Fund);
  - Providing the services to the healthcare providers in the scope of CCMI, DRG value: E10, E11, E12, E12G, E15, E16, E17G, E23G, E24G, E26 and E29 is corrected with the use of the correction factor of 1.1.
(g) In the case of provision of services within the SCS-Infarction within the groups:
  - E23G, E24G, E26, E29, E04, E05, E06, E32, E33, E34 and E36, the value of settlement products is corrected with the use of the correction factor of 1.085;
(h) For healthcare providers providing the services within the CCMI, in which the share of hospitalised patients with diagnosed acute MI treated within the CCMI is at least 60% of all hospitalised patients with this diagnosis, the value of products for settlement of hospitalisation in this healthcare provider (settled with the groups E10, E11, E12G and E15) is corrected with the use of the correction factor of 1.02;
(i) For healthcare providers, in which the share of the patients who received all services specified in the individual treatment plan due to the CCMI assumptions is at least 70% (excluding deaths) of all patients covered with CCMI, the value of products for settlement of hospitalisation (settled with groups E10, E11, E12G and E15) within the CCMI is corrected with the use of the correction factor of 1.03 [10].

## 7. Quality Parameters of the Programme Evaluation

Monitoring and evaluating the CCMI cover both the indicators related to the quality of patient care at the level of the facility implementing the CCMI and the indicators related to treatment outcomes. The parameters used to assess the effects of comprehensive care in the patients after MI after 12 months of care, based on reporting data sent by the healthcare providers to the IT system of the NHF, include:

(1) Percentage share of the patients after MI covered with CCMI;
(2) Percentage share of the patients who received the enter individual treatment plan;
(3) Percentage share of re-hospitalisations for cardiovascular reasons;
(4) share of the patients who have undergone the coronary angioplasty procedure within the second stage of revascularisation;
(5) Share of the patients with ICD;
(6) Share of the patients with implanted cardioverter/defibrillator of CRT-D type;
(7) Share of the patients with implanted S-ICD;
(8) Percentage share of deaths after cardiovascular episodes within:
  - 6 months from MI;
  - 12 months from MI.

In addition, the facility provides the service measures and assesses the following indicators:

(1) Share of the patients who received complete cardiac rehabilitation and reasons for withdrawal from cardiac rehabilitation [%];
(2) Share of the patients who received complete revascularisation and the reasons for failure to provide complete revascularisation;
(3) Share of the patients with left ventricle EF <35%, with implanted relevant
(4) ICD or CRT-D system and the reasons for failure to perform implantation;
(5) Share of the patients who quit smoking [%]–confirmed by a test;
(6) Share of the patients with LDL <1.8 mmol/L [<70 mg%] [%];
(7) Share of the patients with blood pressure <140/90 mmHg [%];
(8) Share of the patients with glycated haemoglobin <7% or fasting blood glucose <7.0 mmol/L [<126 mg%] [%];
(9) Share of the patients with BMI <30 kg/m^2^ [%].

## 8. Primary Prevention

Taking measures to decrease morbidity and premature mortality from CVDs such as MI, heart failure, and cerebrovascular accidents is the priority for the Minister of Health [18]. The systemic measures taken to promote healthy attitudes within primary prevention may include improved diet and physical activity, operation of the National Centre for Nutrition Education or measures taken in health promotion in workplaces [19].

The prevention of CVDs includes the health policy programme entitled: Programme for prevention and control of cardiovascular diseases POLKARD 2017–2020 [20] and National prevention programme for arteriosclerosis and cardiac diseases by education of persons with increased cardiovascular risk factors KORDIAN, aiming at decreasing mortality from CVDs in Poland [21]. The programme continues the previous editions and is dedicated to medical entities involved in diagnosing and treating CVDs and persons at risk of CVDs.

**Figure 5:**
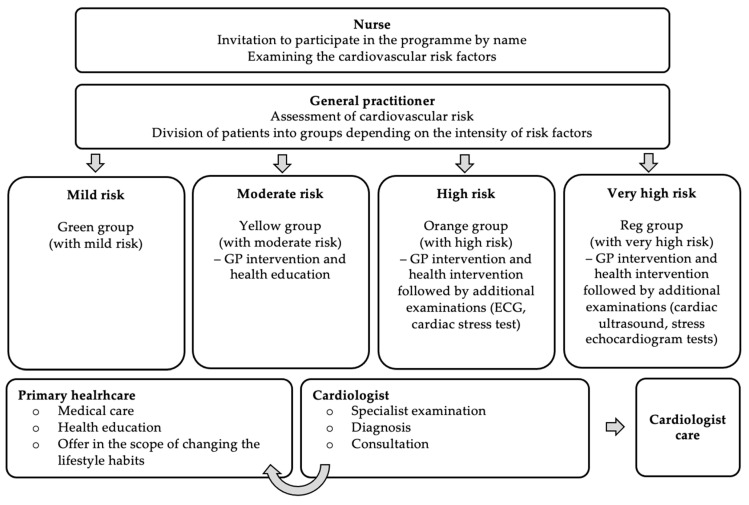
Scheme of programme procedures (2004 version).

As part of primary prevention, a program for the prevention and early detection of CVDs has been implemented since 2004 and still exists today as part of primary healthcare. As indicated in 2004, the identified cardiovascular risk factors include hypertension, lipid disorders, tobacco smoking, low physical activity, overweight and obesity, carbohydrate disorders, poor diet, age and male sex, which is why the primary objective of the programme was to reduce the morbidity and mortality from CVDs by 20%. The programme covered Polish people aged 35–55 (see Figure 5).

It should be pointed out that due to the specialist nature of MI treatment, this treatment, on principle, is carried out by specialist centres. In most regions in Poland, there is accessibility to county hospitals, which, after initial provision, can transfer the patient to the appropriate specialist centre. However, it should be noted that medical emergency teams transporting patients with diagnosed MI generally choose specialised centres. In the authors’ opinion, the number of cardiac rehabilitation centres could be increased; however, considering the limited availability of medical staff, this issue may not be solved in the near future.

The expected effect of the implemented prevention programme was decreasing morbidity and mortality from CVDs, decreasing frequency and intensity of cardiovascular risk factors in the programme participants, early identification of persons with increased global coronary risk and implementation of preventive measures in such persons. This programme also improved the competencies of medical personnel (nurses and physicians) in the area of primary prevention and early detection of cardiovascular risks.

Since 2009, the actions taken under the prevention program have been included in the catalogue of guaranteed benefits under primary healthcare. The NHF President specified the terms and conditions of implementing the contracts in this scope [22,23]. Guaranteed services provided by the general practitioner include medical consultation in outpatient conditions in direct contact with the healthcare recipient, or remotely by using the ITC or communications systems; medical consultation provided at the home of the healthcare recipient in medically justified cases; services within the CVDs prevention. The eligibility criteria for guaranteed services within CVDs prevention cover the persons with the following risk factors:

(1) Hypertension (RR > 140/90 mmHg);
(2) Lipid disorders (increased total blood cholesterol, LDL-cholesterol, triglycerides and low LDL-cholesterol level);
(3) Tobacco smoking;
(4) Low physical activity;
(5) Overweight and obesity;
(6) Impaired glucose tolerance;
(7) Increased fibrinogen level;
(8) Increased uric acid level;
(9) Excessive stress;
(10) Poor diet;
(11) Age;
(12) Male sex;
(13) Henetic loads listed by the general practitioner at the healthcare provider’s and in a given calendar year, covered with the healthcare service contract, aged 35, 40, 45, 50 and 55, with no diagnosed CVDs and receiving no services provided within the CVDs prevention within the last five years (including in the other healthcare providers).

## 9. The Required Medical Procedures for Cardiovascular Diseases

(1) Medical history and completing the preventive check-up report;
(2) Blood biochemical tests (total blood cholesterol, LDL-cholesterol, HDL-cholesterol, triglycerides and blood glucose levels), blood pressure measurement, body mass index (BMI) determination;
(3) Scheduling the visit at the general practitioner holding the list with the name of this patient;
(4) Entering the test results into the prevention check-up report;
(5) During the visit at the general practitioner:
  (a) Examination of healthcare service recipient and assessment of cardiovascular risk factors;
  (b) Classification of the patient into the respective risk group and assessment of the global risk of the cardiovascular incident in future according to SCORE and entering the obtained score in the preventive check-up report;
  (c) Health education of the patient and decision on further management.
(6) Providing information on examination results by the general physician to the patient with diagnosed CVD and giving recommendations on the need to change lifestyle habits or referring the patient to further diagnosing or treatment.

## 10. Conclusions and Recommendations on Health Policy

Since autumn 2017, the pioneer CCMI model has been operating in Poland. This programme covers intensive treatment, rehabilitation and follow-up in outpatient conditions. The development and improvement of each stage followed the ESC guidelines. The programme aimed to improve the quality of care, prevention and, primarily, better long-term outcomes for patients.

Confirmed benefits from the programme require clinical data covering a significant percentage of the population and long-time of data collection. Nonetheless, the results obtained at the early stage of the model are promising. The effectiveness of the described CCMI model was demonstrated in our recent study from 2022: Survival analysis of patients with acute coronary syndrome receiving comprehensive and coordinated care after myocardial infarction (KOS-Infarction) [8]. The study indicated that the CCMI program reduces the risk of death by 29% in the first year after MI. According to the study, the implementation of early cardiac rehabilitation and ongoing access to a cardiac specialists are the direct causes of the reduction in death. In addition, we observed improved quality of life and lower risk of subsequent cardiac incidents in the patients covered with the programme (the number of secondary rehabilitations was substantially decreased). Moreover, it was revealed that 10% of patients being treated for the CCMI program were certified as being able to work (return from sickness benefits due to myocardial infarction within four months).

Total benefits that may directly affect the decrease in the number of deaths in Poland from cardiovascular reasons and the burden of the healthcare system related to the treatment of subsequent cardiac episodes are invaluable. The profits will include both the patients and the healthcare system. Despite the continuous improvement of the programme and introducing the solutions aiming to promote growth trends among the healthcare providers and recipients, there are still discrepancies that result in low participation in the programme among the persons who could benefit from it.

The programme operation should be expanded in the regions with the lowest share of facilities covered with CCMI. The Polish Cardiac Society points out the need to cover patients treated outside the facilities from the hospital network with the programme. The programme of coordinated specialist care after MI provides a better quality of life, which implies considering a systemic combination of the existing primary prevention programme into the CCMI module. Additionally, the currently implemented pilot cardiology network course requires in-depth monitoring and detailed analyses.

## Data Availability

The authors confirm that all data underlying the findings described in this manuscript is fully available to all interested researchers upon request.
